# Monte Carlo simulation of linac using PRIMO

**DOI:** 10.1186/s13014-022-02149-5

**Published:** 2022-11-16

**Authors:** Yang Li, Xingru Sun, Ying Liang, Yuchao Hu, Chenbin Liu

**Affiliations:** Department of Radiation Oncology, National Cancer Center/National Clinical Research Center for Cancer/Cancer Hospital & Shenzhen Hospital, Chinese Academy of Medical Sciences and Peking Union Medical College, Shenzhen, 518116 China

**Keywords:** Monte Carlo, Percentage depth dose, Off axis ratio, Transmission factor, Dose leaf gap

## Abstract

**Background:**

Monte Carlo simulation is considered as the most accurate method for dose calculation in radiotherapy. PRIMO is a Monte-Carlo program with a user-friendly graphical interface.

**Material and method:**

A VitalBeam with 6MV and 6MV flattening filter free (FFF), equipped with the 120 Millennium multileaf collimator was simulated by PRIMO. We adjusted initial energy, energy full width at half maximum (FWHM), focal spot FWHM, and beam divergence to match the measurements. The water tank and ion-chamber were used in the measurement. Percentage depth dose (PDD) and off axis ratio (OAR) were evaluated with gamma passing rates (GPRs) implemented in PRIMO. PDDs were matched at different widths of standard square fields. OARs were matched at five depths. Transmission factor and dose leaf gap (DLG) were simulated. DLG was measured by electronic portal imaging device using a sweeping gap method.

**Result:**

For the criterion of 2%/2 mm, 1%/2 mm and 1%/1 mm, the GPRs of 6MV PDD were 99.33–100%, 99–100%, and 99–100%, respectively; the GPRs of 6MV FFF PDD were 99.33–100%, 98.99–99.66%, and 97.64–98.99%, respectively; the GPRs of 6MV OAR were 96.4–100%, 90.99–100%, and 85.12–98.62%, respectively; the GPRs of 6MV FFF OAR were 95.15–100%, 89.32–100%, and 87.02–99.74%, respectively. The calculated DLG matched well with the measurement (6MV: 1.36 mm vs. 1.41 mm; 6MV FFF: 1.07 mm vs. 1.03 mm, simulation vs measurement). The transmission factors were similar (6MV: 1.25% vs. 1.32%; 6MV FFF: 0.8% vs. 1.12%, simulation vs measurement).

**Conclusion:**

The calculated PDD, OAR, DLG and transmission factor were all in good agreement with measurements. PRIMO is an independent (with respect to analytical dose calculation algorithm) and accurate Monte Carlo tool.

**Supplementary Information:**

The online version contains supplementary material available at 10.1186/s13014-022-02149-5.

## Background

Monte Carlo (MC) method was considered as the “golden standard” method to perform absorbed dose calculations in external radiotherapy [1–8]. A number of general-purpose MC codes were developed such as FLUKA [9], MCNP [10, 11], EGSnrc [12], PENELOPE [13], GEANT [14, 15], and employed for research and development in medical applications during the past decades. The state-of-the-art Monte Carlo method was available in a few commercial treatment planning systems (TPS) such as Elekta Monaco XVMC (X-ray Voxel MC) [16], Accuray Multiplan [17], Brain Lab IPlan [18], Nomos Corvus [19], which fully takes the electron transport in the medium/water into account and achieves highest accuracy. Despite a continuously increasing interest of MC in radiotherapy treatment planning, the introduction of MC algorithms in clinical practice has been delayed by the excessive calculation time involved.

Typically, the accurate Monte Carlo simulation needed the detailed description of the material properties and geometrical model of the linac head, which was a time-consuming and error-prone task [20]. PRIMO is a program based on PENELOPE 2011 [13, 21, 22], PENEASY [23], dose planning method (DPM) [24], and PENEASYLINAC [23]. It has a graphical user interface and encompasses all the components in a single user-friendly environment. Furthermore, PRIMO is an open access software and everyone can get its license for free.

There were many studies about linac simulation using PRIMO [25–39]. Different types of linac machines were simulated using PRIMO, including Varian TrueBeam [25, 29–31, 35, 37, 39], Varian 2100 C/D [27], Clinac 2300 [28], Clinac® iX [33, 36], Trilogy [32], Novalis [32], EDGE [34]. Some of the previous studies adopted the phase space files (PSF) from MyVarian website to model the linac head [25, 29, 35, 39]. Some researchers simulated linacs using the default beam parameters given by PRIMO [31, 34], while other studies modified the beam parameters based on the default recommendations in PRIMO [27, 28, 30, 32, 33, 36]. As two critical parameters of linac head model, percentage depth dose (PDD) and off-axis ratio (OAR) were evaluated in many studies. Belosi et al. [25] studied the accuracy of Varian PSF for flattening filter free (FFF) beams, and compared to measured PDD and OAR from ten TrueBeam linacs. Pita et al. [28] simulated a Varian Clinac 2300, compared the PDD and OAR with the measurements, and validated the static field and IMRT field.

Previous studies using PRIMO focused on the evaluation of linac head model [25–27, 30, 31, 35, 36, 38]. However, the evaluation of multileaf collimator (MLC) model was critical in clinical use [40]. Since the deviation of MLC model in Eclipse™ TPS (transmission factor and DLG) could produce large dosimetric discrepancies, the MLC model should be evaluated comprehensively in beam configuration [40]. Paganini et al. [34] established a series of different MLC patterns to evaluate the MLC model. The difference of DLG values obtained with PRIMO, Acuros, and EBT3 Gafchromic film measurements were within 0.008 cm [34]. Although the film has high spatial resolution, it has the issues of regional non-uniformity and positioning error, which may cause the measurement uncertainty [41]. In this study, we introduced EPID measurement in the evaluation of MLC model, which allows fast measurement, less positioning error, and highly reproducible setup. As far as we know, there were no previous reports about the comparison of simulated DLG and transmission factor with EPID measurements.

In this study, we investigated the linac head and MLC model of Varian VitalBeam™, including PDD, OAR, transmission factor and DLG. Firstly, the linac head with photon beams of 6MV and 6MV FFF was simulated in PRIMO. The initial energy, energy full width at half maximum (FWHM), focal spot FWHM, and beam divergence were tuned in PRIMO to match the PDD and OAR measurement. Secondly, we simulated the transmission factor and DLG in the Millennium 120 MLC model. The simulated results were compared with the EPID measurement and optimized value using clinical treatment plans.

## Material and method

### Measurement equipment

All measurements were conducted on a Varian VitalBeam™ (Varian Medical Systems, Palo Alto, CA) linac equipped with a Millennium 120 leaf MLC. Photon beams of 6MV and 6MV FFF were used. A PTW BEAMSCAN water phantom (PTW Freiburg, Germany), an ion chamber (Semiflex 3D Type TW31021, PTW, Freiburg, Germany), and an electrometer (PTW UNIDOS E, T10009, PTW, Freiburg, Germany) were used in the measurements. The commercial software MEPHYSTO™ (PTW, Freiburg, Germany) was used to perform the evaluation of dose profiles. PRIMO software (version 0.3.64.1800_x64) was installed on a workstation (Windows 10 Enterprise, Intel Core i7-7820X CPU@3.6 GHz, RAM 32 GB).

Amorphous silicon-based electronic portal imaging device (EPID) was used to measure the transmission factor and DLG. The active area of EPID detector was 30 cm × 40 cm (1024 × 768 matrix) with pixel size of 0.39 mm. The images were acquired in “the integrated image” mode used 4DTC as the acquisition workstation at the source detector distance (SDD) of 105 cm. The frame rate of EPID is 9.6 frames/s. Portal dose image prediction (version 8.0, Varian Medical Systems, Inc., USA) was used to compare the predicted portal dose images with the measured ones. Dose image was calculated using the Portal Dose Image Prediction (PDIP) algorithm in the Eclipse™ version 13.6.23 (Varian Medical Systems Inc., Palo Alto, CA).

The dosimetric leaf gap (DLG) in Eclipse™ TPS was optimized to reduce the discrepancies between the measured dose distributions and the calculated ones of patient treatment plans. The Delta4 phantom system (Scandidos, Uppsala, Sweden) was used in the dose verification for the clinical treatment plans. Gamma-index evaluation method implemented in Delta4 was applied to quantify the discrepancies of dose distributions. Delta4 is a cylindrical polymethyl-methacrylate phantom with 22 cm in diameter and 40 cm in length. The mass density of the phantom was 1.19 g/cm^3^, and the relative electron density was 1.147. It consists of 1069 p-type Silicon diodes in a crossed array inside the phantom, with 5 mm resolution in central area and 10 mm resolution in the peripheral area. The software ScandiDos Delta4 allowed the users to compare the measured dose distribution with the dose distribution calculated using TPS.

### Measurements of the critical parameters for TPS simulation

We measured the PDD and OAR of 6MV and 6MV FFF photon beam for Varian VitalBeam linac at Chinese Academy of Medical Sciences Cancer Hospital, Shenzhen center, respectively. The scan range of the detector in horizontal plane was 50 cm by 50 cm. The measurements were performed in continuous scanning mode at the speed of 10 mm/s. Different square fields (3 cm, 4 cm, 6 cm, 8 cm, 10 cm, 15 cm, 20 cm, 30 cm, and 40 cm) were used with a fixed source-to-phantom distance (SPD) of 100 cm.

**Fig. 1:**
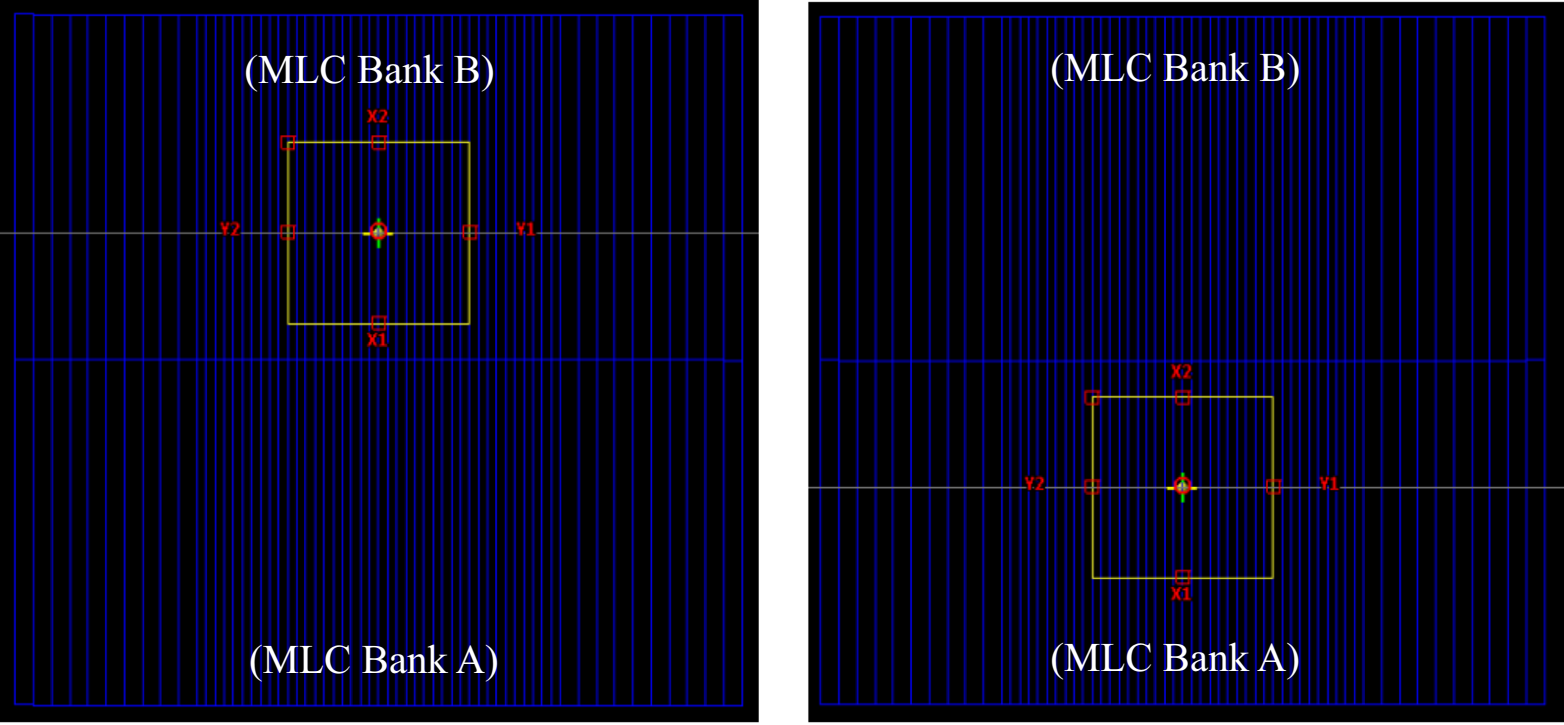
The BEV projection of the completely blocked MLC field with MLC bank A closed (R_A_) and bank B closed (R_B_), respectively

Transmission factor and DLG are two crucial parameters to model the rounded MLC leaf in Eclipse™ TPS. The transmission factor is determined by the material and height of the MLC leaf, beam quality, and etc. EPID was used to measure the MLC transmission in two MLC-closed fields: one with MLC bank A closed and the other with MLC bank B closed as shown in Fig. 1. The transmission factor was calculated using the following equations:

1 $$tr\left( A \right) = R_{A} /R_{open}$$

2 $$tr\left( B \right) = R_{B} /R_{open}$$

3 $$R_{T} = \left[ {\frac{tr\left( A \right) + tr\left( B \right)}{2}} \right]$$

where *tr*(*A*) and *tr*(*B*) are transmission factors of MLC bank A and bank B, respectively. *R*_*open*_ is the reading of the open field. *R*_*A*_ and *R*_*B*_ are the readings of closed MLC bank A and closed MLC bank B, respectively. *R*_*T*_ is the average MLC leaf transmission for both MLC banks.

**Fig. 2:**
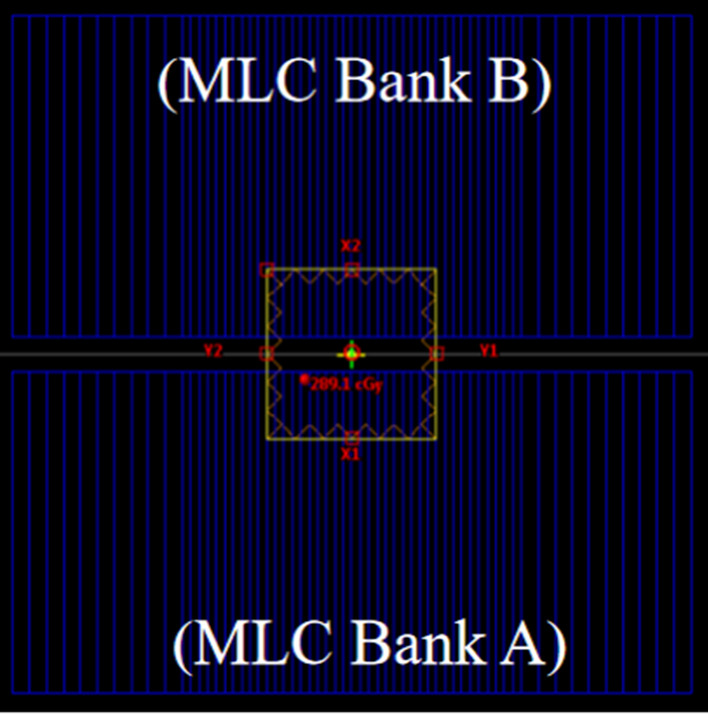
DLG test plan with gap width of 20 mm

DLG was measured using sweeping gap technique [42]. The sweeping gap test plans were designed in Eclipse™ TPS (Varian Medical Systems, Inc., USA), including dynamic sweeping gaps of 2 mm, 4 mm, 6 mm, 10 mm, 14 mm, 16 mm, and 20 mm (shown in Fig. 2), two closed-MLC fields, and one open-MLC field. The test plans were generated with gantry angle of 0 degree, collimator setting of 90 degree, SSD at 100 cm, MLC moving from − 6 to + 6 cm in dynamic mode at the speed of 2.5 cm/s. For each treatment plan, the jaws were set to 10 cm by 10 cm as the reference field. Each sweeping gap traveled across the reference field, and the delivered dose was 100 MU.

In order to evaluate the contribution of sweeping gap field, the MLC transmission reading *R*_*gT*_ was subtracted from the initial ionization reading $\cdot R_{g}$. The corrected gap reading (${R}_{{g}^{^{\prime}}}$) and *R*_*gT*_ for each gap (*g*) were defined by Varian guideline as:

4 $$R_{gT} = R_{T} \cdot \left[ {1 - \frac{g}{120}} \right]$$

5 $$R_{{g^{\prime}}} = R_{g} - R_{gT}$$

where *g* is the nominal gap width (unit: mm), the sweeping gap movement range is 120 mm. *R*_*g*_ is the initial sweeping gap field reading (CU values for EPID). The corrected gap readings were fitted using linear regression method. DLG was the value at the intersection between the extrapolated extension of the fitted line and y-axis (Fig. 6). R-squared was used to measure the goodness-of-fit for the linear regression model.

### PRIMO simulation

**Fig. 3:**
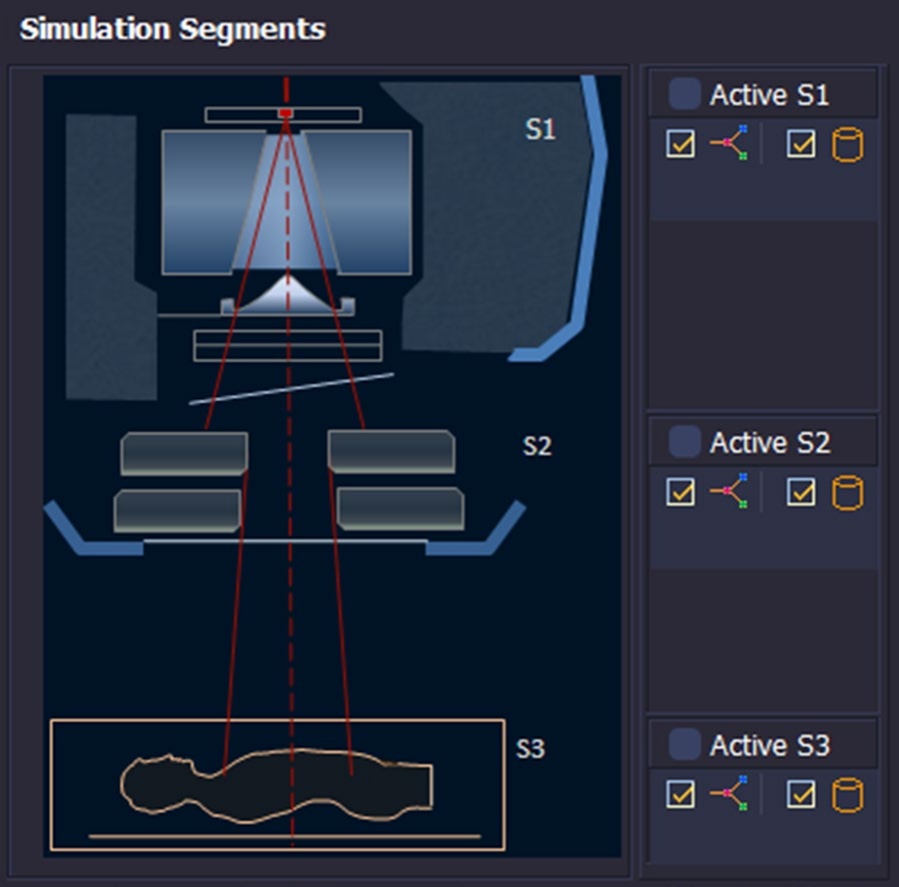
The three segments in PRIMO simulation

The PRIMO simulation setup consists of three segments [31, 32, 43] as shown in Fig. 3. In segment 1 (s1), the upper part of the linac was simulated. The user could adjust the primary beam parameters including nominal energy, initial energy, energy FWHM, focal spot FWHM, and beam divergence. In s1, the four parameters were tuned to match the PDDs in different field size and OARs in different depths. In the segment 2 (s2), the field parameters were edited, including the treatment technique, beam weights, gantry start and end angles, collimator angle, couch angle, MLC type, aperture size, applicator, and isocenter location. In segment 3 (s3), the linac model was applied in patient and phantom geometry. The three segments can be grouped in a user-defined way [43].

**Fig. 4:**
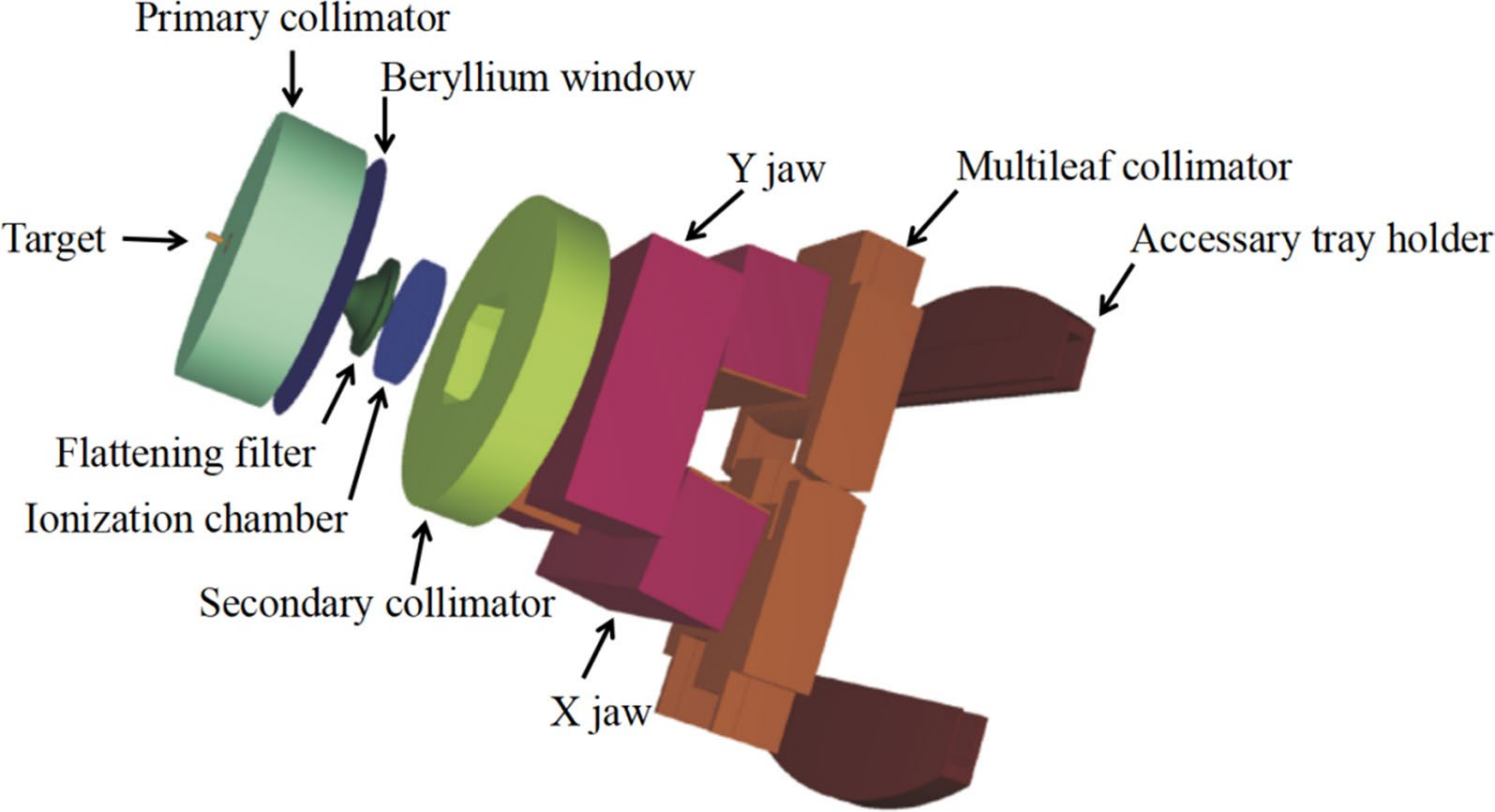
The illustration of head geometry for Varian VitalBeam™ operating in photon mode (From PRIMO user’s manual software version 0.3.(32–64).1880)

In PRIMO, Varian 2100 linac model was recommended to simulate TrueBeam for 6MV, while FakeBeam linac model is an experimentally based geometry of TrueBeam for 6MV FFF and 10MV FFF. In segment 1, we selected Varian 2100 and Fake Beam from PRIMO linac model list, as the initial models of 6MV and 6MV FFF Varian VitalBeam linac, respectively. The linac head geometry is shown in Fig. 4. The transport parameters of linac head components are shown in the Additional file 1: Table 1. To determine the beam characteristics, there were four crucial parameters in PRIMO, including initial electron energy, energy FWHM, FWHM of the focal spot size, and beam divergence. We fine-tuned the four crucial parameters to match the simulated PDD and OAR with our measurements. Among the four parameters, the initial electron energy was firstly determined based on the depth of the maximum dose on the depth dose curves based on field sizes of 10 cm × 10 cm. The other three parameters were adjusted until both simulated PDD and OAR had the highest GPRs compared with our measurements in all the considered field sizes (For field sizes smaller than 10 cm × 10 cm (included), the GPRs (1%/1 mm) were higher than 90%, other field sizes were higher than 85%).

In our study, the search range of initial electron energy was 5.4–6.6 MeV, and the interval was 0.1 MeV. The energy FWHM varied from 0 to 0.1 MeV with an interval of 0.01 MeV. The FWHM of the focal spot size varied from 0 to 0.5 cm with an interval of 0.05 cm. The divergence of the parallel beams was considered to be zero degree.

In segment (s1), the PENELOPE computation engine was used to obtain the phase-space file. Splitting roulette was adopted as the variance reduction technique to improve simulating efficiency. In segment 2 (s2) and segment 3 (s3), the dose planning method (DPM) was employed to calculate dose distribution. The splitting factor in DPM was 300. Particle histories increased from 10^9^ in s1 to 10^10^ in s2 and s3. The number of particle histories increased along with the increase of the field size to reduce the uncertainty.

Both PDD and OAR were simulated in the water phantom under the following condition: gantry angle, 0 degree; collimator angle, 0 degree; SSD, 100 cm; fixed bin size in the tallying volume, 0.2 × 0.2 × 0.2cm^3^. In our study, there were nine square field sizes considered, including 3 cm, 4 cm, 6 cm, 8 cm, 10 cm, 15 cm, 20 cm, 30 cm, and 40 cm. All the square fields were collimated by the jaws. The tallying volumes increased with the increase of the square field size. The corresponding tallying volumes in PRIMO were 9 × 9 × 35cm^3^, 12 × 12 × 35cm^3^, 18 × 18 × 35cm^3^, 24 × 24 × 35cm^3^, 30 × 30 × 35cm^3^, 40 × 40 × 35cm^3^, 50 × 50 × 35cm^3^, 60 × 60 × 35cm^3^, and 70 × 70 × 35cm^3^, respectively.

**Fig. 5:**
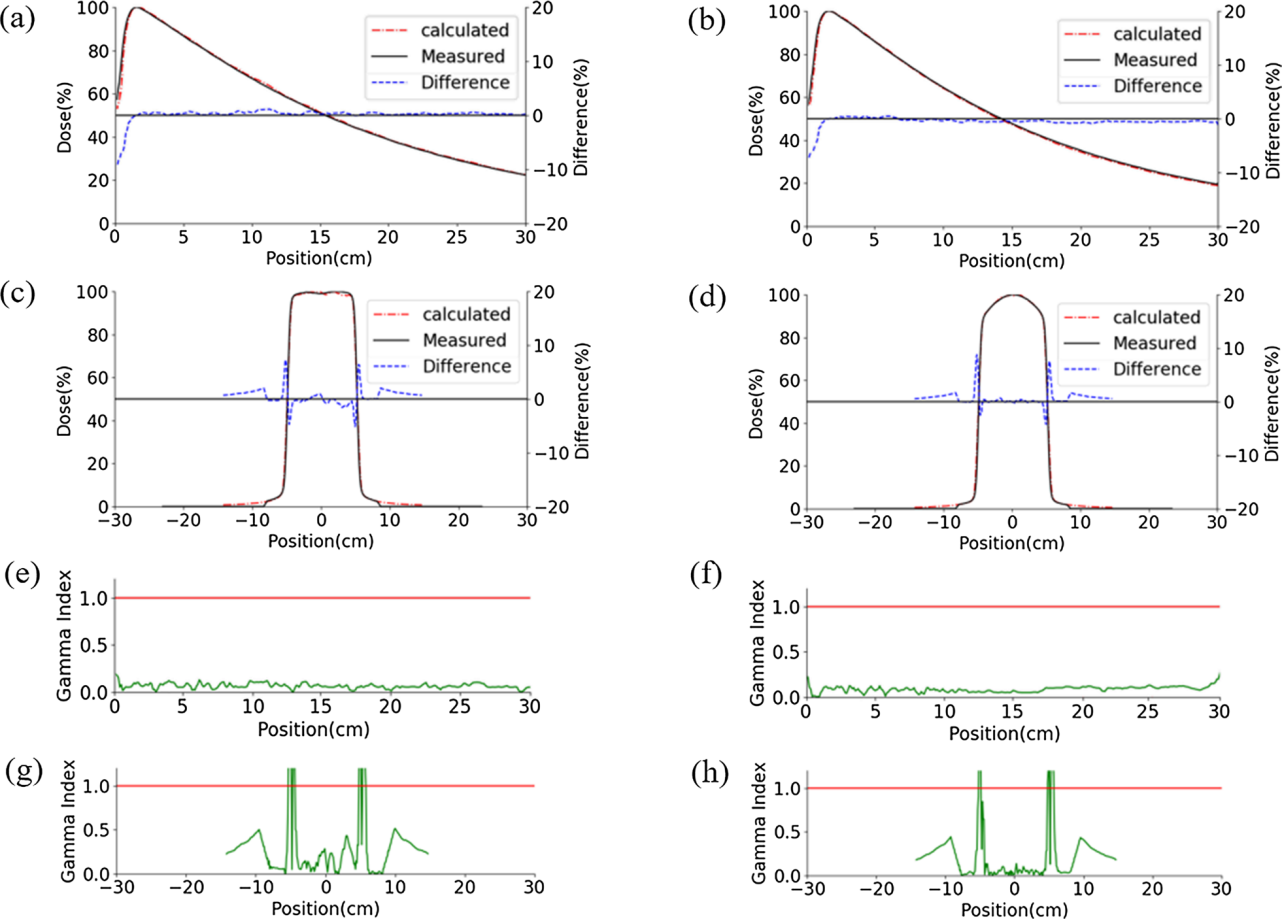
Comparison between simulations and measurements of PDD and OAR (The dose distribution was normalized to the maximum dose point). **a** PDD 6MV, 10 × 10 cm^2^, **b** PDD 6MV FFF, 10 × 10 cm^2^, **c** the gamma index for 6MV PDD, **d** the gamma index for 6MV FFF PDD, **e** OAR 6MV, 10 × 10 cm^2^ depth at 1.5 cm, **f** OAR 6MV FFF, 10 × 10 cm^2^ depth at 1.5 cm, **g** the gamma index for 6MV OAR, **h** the gamma index for 6MV FFF OAR

The model of Millennium 120 MLC was selected in PRIMO to evaluate the MLC model. The model consists of 40 central leaf pairs with a 5 mm projection width at the isocenter (SSD = 100 cm) and 20 outer leaf pairs with a width of 10 mm. The MLC transmission factor were obtained using Eqs. (1)–(3). The designed sweeping gap test plans were imported into PRIMO. The dose tallying volume in simulation was 15 × 15 × 10cm^3^. The point dose (R_g_) was acquired at 5 cm below the dose tallying volume. The R_g_ readings of different sweeping gap test plans were fitted using linear regression method. DLG was acquired as the value at the intersection of the extrapolated fitting line and y-axis (Fig. 5).

## Results

### PDDs and OARs of different field sizes

For PDD and OAR, we modified the critical beam parameters to match the simulation with measurement results. The critical parameters of 6MV photon beam were listed as follows: initial electron energy, 5.9 MeV; FWHM, 0.03 MeV; focal spot FWHM, 0.1 cm; beam divergence, 0 degree. The critical parameters of 6MV FFF photon beam were listed as follows: initial electron energy, 5.8 MeV; FWHM, 0.04 MeV; focal spot FWHM, 0.20 cm; beam divergence, 0 degree. The average statistical dose uncertainty in the PRIMO simulation was below 2%. All uncertainties reported by PRIMO are given at 2 standard deviations (2σ).

The comparison of measured and simulated PDD and OAR were shown in Fig. 5. The GPRs for PDD and OAR were shown in the Additional file 1: Tables 2, 3, 4 and 5, respectively. For 6MV photon beam PDD, the GPRs were 100% in all the considered fields as shown in the Additional file 1: Table 2, when the 3%/3 mm gamma criterion was used. The GPRs of PDD in all the nine field sizes were over 99.33%, 99% and 99% with the criteria of 2%/2 mm, 1%/2 mm and 1%/1 mm, respectively.

For 6MV FFF photon beam PDD, the GPRs were 100% in all the considered fields using the criteria of 3%/3 mm. As shown in the Additional file 1: Table 3, the GPRs of PDD in the nine field sizes were over 99.33%, 98.99% and 97.64% with the criteria of 2%/2 mm, 1%/2 mm and 1%/1 mm, respectively.

For 6MV photon beam OAR, the GPRs were 100% in all the nine fields with the criteria of 3%/3 mm. As shown in the Additional file 1: Table 4, the GPRs of OAR in the nine field sizes were over 96.4%, 90.6%, and 85.12% with the criteria of 2%/2 mm, 1%/2 mm and 1%/1 mm, respectively.

For 6MV FFF photon beam OAR, the GPRs were 100% in all the nine fields with the criteria of 3%/3 mm. As shown in the Additional file 1: Table 5, the GPRs of OAR in the nine field sizes were over 95.15%, 89.32%, and 86.55% with the criteria of 2%/2 mm, 1%/2 mm and 1%/1 mm, respectively.

### Transmission factor and DLG

The transmission factor for 6MV photon beam was 1.25% versus 1.32%, EPID measured vs PRIMO simulated. For 6MV FFF photon beam, the transmission factor was 0.8% versus 1.12%, EPID measured vs PRIMO simulated (Table 1).

**Table 1:** The transmission factor and DLG values for 6MV and 6MV FFF photon beams

		6MV	6MV FFF
Transmission factor (%)	EPID measured	1.32	1.12
	PRIMO simulated	1.25	0.80
DLG (mm)	EPID measured	1.41	1.03
	PRIMO simulated	1.36	1.07
	Optimized	1.45	1.36

See more details of the optimized DLG in supplemental material

**Fig. 6:**
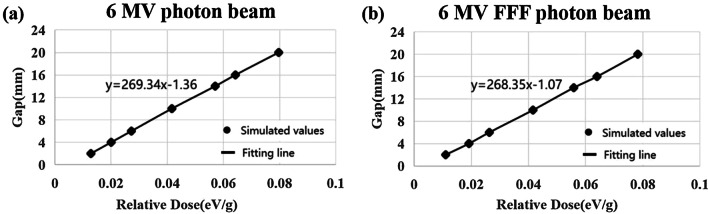
DLG obtained from PRIMO simulation. **a** 6MV photon beam, **b** DLG obtained using PRIMO for 6MV FFF photon beam (R^2^ = 0.999)

For 6MV photon beam, the linear regression equation based on PRIMO simulation was y = 269.34x − 1.36 with R square of 0.99. The slope of the fitted line is 269.34, and the DLG value was 1.36. For 6MV FFF photon beam, the linear regression equation based on PRIMO simulation was y = 268.35x − 1.07 with R square of 0.99 (Fig. 6). The slope of the fitted line is 268.35, and the DLG value was 1.07.

**Fig. 7:**
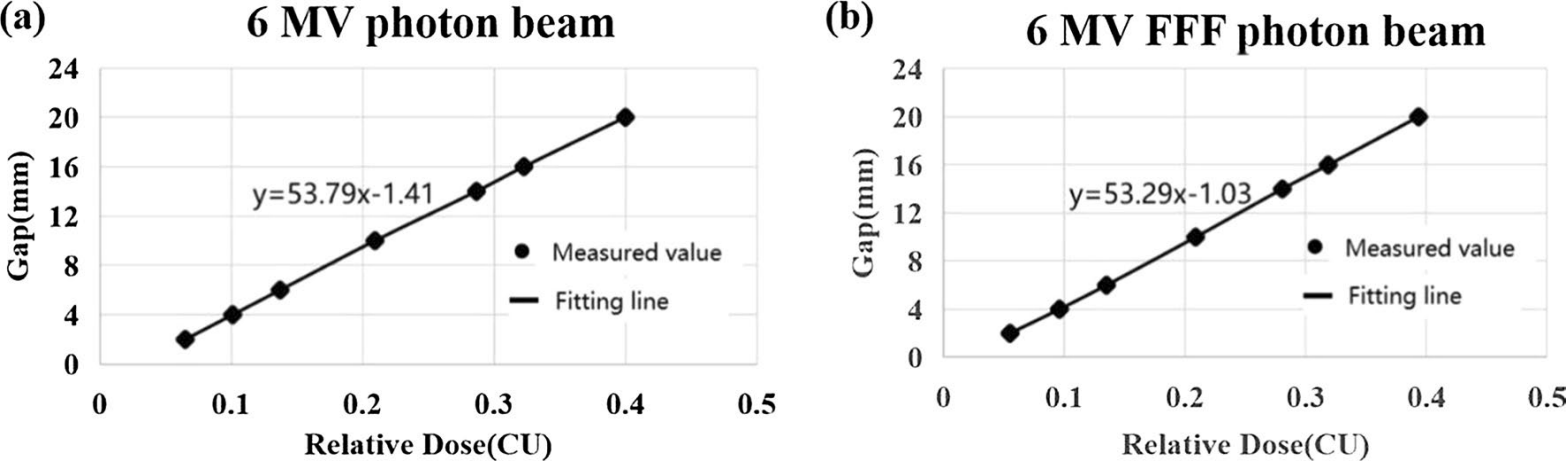
DLG obtained from EPID measurements. **a** 6MV photon beam, **b** 6MV FFF photon beam (R^2^ = 0.999)

For 6MV photon beam, the linear regression equation based on EPID measurements was y = 53.79x − 1.41 with R square of 0.99. The slope of the fitted line is 53.79, and the DLG value was 1.41. For 6MV FFF photon beam, the linear regression equation based on EPID measurements was y = 53.29x − 1.03 with R square of 0.99 (Fig. 7). The slope of the fitted line is 53.29, and the DLG value was 1.03.

## Discussion

In this study, we tuned the four crucial parameters in PRIMO, including initial energy, energy FWHM, focal spot FWHM, and beam divergence. The fine-tuned Monte Carlo program was used to verify linac head model and MLC model. PDD and OAR are two vital parameters of linac head model. Transmission factor and DLG are the important parameters of MLC model in Eclipse™ TPS. Nine field sizes were considered, including 3 cm, 4 cm, 6 cm, 8 cm, 10 cm, 15 cm, 20 cm, 30 cm, and 40 cm. The results demonstrated that the simulated PDD, OAR, transmission factor, and DLG matched well with the measurements.

Although no programming was required when using PRIMO, the PDD and OAR match in beam commissioning of the linac head model was intensive and laborious. Some studies used the default parameters in the primary beam setup segment [31, 34]. Some research groups imported the external phase space files offered by Varian into PRIMO to reduce the workload [25, 29, 35, 39]. In this study, we tuned the parameters in the primary beam setup segment to match our measurements of the linac machines. Many studies about beam configuration for linac model only compared simulated PDD and OAR with measurements [25, 26, 29, 30, 38, 39]. It could lead to dose deviation due to the lack of MLC model configuration when clinical treatment plans were compared with simulation results. In this study, MLC modeling combined with PDD and OAR matching covered a more integrated and accurate work. The fine-tuned linac models using PRIMO have potential to be used as an independent tool in quality assurance of clinical treatment plans.

There are mainly three methods to measure the transmission factor and DLG, including ion-chamber, film and EPID [44]. Due to the independence of the energy spectrum and linear increase with dose, the ion chamber measurements were considered as the gold standard [45]. However, the positioning error and the volume effect may result in measurement error in the condition of small MLC gaps. Paganini studied the DLG and transmission factor of 10MV FFF photon beam using PRIMO and compared the results with the microdiamond detector and Gafchromic film measurements [34]. Film has the advantage of high spatial resolution and reproducibility, but the non-uniform response and data analysis may increase the measurement uncertainty. Compared with ion chamber and film, EPID has the advantage of fast measurement, easy operation, less positioning error, and highly reproducible set-up [45]. In this study, EPID was used to measure the transmission factor and DLG.

Some studies found it could generate large difference between calculated and measured dose distributions when using the DLG obtained by sweeping gap method. It is recommended to introduce the optimized DLG using clinical treatment plans [46, 47]. In this study, we optimized the DLG value using Delta4 phantom and 100 clinical treatment plans. The optimized DLG produced the highest GPRs between the calculated and measured dose distributions in the clinical treatment plans. We found the optimized DLGs were similar with the measured ones, and larger than the simulated ones, which is consistent with the previous study [48]. Our result demonstrated that the initial values of transmission factor and DLG can be obtained using PRIMO. In the future, we can generate transmission factor and DLG based on the calculation using PRIMO and fine-tune the two parameters using patient specific quality assurance measurements. This will help the future commissioning of Eclipse in new linac site.

As an independent dose verification software, PRIMO has potential to be used in clinical application, such as beam commission, treatment planning verification, and quality assurance. Our future work will focus on the clinical applications of the commissioned PRIMO program, such as the treatment planning calculations of skin doses with bolus, and the verification of dose distributions in the inhomogeneous medium.

## Conclusion

This study used the PRIMO software to simulate the photon beam 6MV and 6MV FFF of a Varian VitalBeam linac. Our study showed that PRIMO is an accurate, self-contained, Monte Carlo-based linac simulator and dose calculator with a user-friendly graphical interface. The commissioned PRIMO can be used as an effective tool to provide a potential independent quality assurance.

## Supplementary Information

**Additional file 1**. The transport parameters configuration in PRIMO and gamma passing rates for PDD and OAR.

## Data Availability

The datasets used during the current study are available from the corresponding author on reasonable request.

## Abbreviations

MC: Monte Carlo
TPS: Treatment planning system
DPM: Dose planning method
PSF: Phase space file
FFF: Flattening filter free
PDD: Percentage depth dose
OAR: Off-axis ratio
MLC: Multileaf collimator
DLG: Dose leaf gap
FWHM: Energy full width at half maximum
EPID: Electronic portal imaging device
SDD: Source detector distance
SPD: Source-to-phantom distance
SBRT: Stereotactic body radiation therapy
PO: Photon optimizer
AAA: Anisotropic analytical algorithm
DD: Dose-difference
DTA: Distance-to-agreement
GPR: Gamma passing rate
IMRT: Intensity modulated radiation therapy
