# Supplementary material for: Monte Carlo simulation of linac using PRIMO
Source: Radiat Oncol. 2022 Nov 16;17:185. doi: 10.1186/s13014-022-02149-5 (PMC9667592; doi:10.1186/s13014-022-02149-5)
Supplement: Supplementary file 1 — Additional file 1. The transport parameters configuration in PRIMO and gamma passing rates for PDD and OAR. [file 13014_2022_2149_MOESM1_ESM.docx]

To determine the tradeoff between speed and accuracy, the transport parameters (Supplementary table 1) were recommended in PRIMO.

Supplementary table 1-The transport parameters configuration in PRIMO.

| Conponent | E_abs_  (e-)  (eV) | E_abs_  (pho)  (eV) | E_abs_  (e+)  (eV) | C_1_ | C_2_ | WCC | WCR | dsMax  (cm) |
| --- | --- | --- | --- | --- | --- | --- | --- | --- |
| Mylar-Mirror | 2x10^5^ | 5x10^4^ | 2x10^5^ | 1x10^-1^ | 1x10^-1^ | 2x10^5^ | 2x10^5^ | 5x10^-4^ |
| W-jaws-skin | 2x10^5^ | 5x10^4^ | 2x10^5^ | 1x10^-1^ | 1x10^-1^ | 2x10^5^ | 2x10^5^ | 1x10^30^ |
| Be-window | 2x10^5^ | 5x10^4^ | 2x10^5^ | 1x10^-1^ | 1x10^-1^ | 2x10^5^ | 2x10^5^ | 2.5x10^-4^ |
| Ta-18x-F, Filter-insert | 2x10^5^ | 5x10^4^ | 2x10^5^ | 1x10^-1^ | 1x10^-1^ | 2x10^5^ | 2x10^5^ | 1.33x10^-1^ |
| Kapton-Ion-Chamber | 2x10^5^ | 5x10^4^ | 2x10^5^ | 1x10^-1^ | 1x10^-1^ | 2x10^5^ | 2x10^5^ | 1.2x10^-4^ |
| Air | 2x10^5^ | 5x10^4^ | 2x10^5^ | 1x10^-1^ | 1x10^-1^ | 2x10^5^ | 2x10^5^ | 5x10^-1^ |
| Cu-Flattening-filter | 2x10^5^ | 5x10^4^ | 2x10^5^ | 1x10^-1^ | 1x10^-1^ | 2x10^5^ | 2x10^5^ | 5x10^-1^ |
| Fe-18X-flattening-filter | 2x10^5^ | 5x10^4^ | 2x10^5^ | 1x10^-1^ | 1x10^-1^ | 2x10^5^ | 2x10^5^ | 1x10^30^ |
| W-MLC-lower-skin | 2x10^5^ | 5x10^4^ | 2x10^5^ | 1x10^-1^ | 1x10^-1^ | 2x10^5^ | 2x10^5^ | 5x10^-3^ |
| W-MLC-frontal-skin | 2x10^5^ | 5x10^4^ | 2x10^5^ | 1x10^-1^ | 1x10^-1^ | 2x10^5^ | 2x10^5^ | 2x10^-2^ |
| W-Primary-coll-non-skin | 9x10^8^ | 5x10^4^ | 9x10^8^ | 0 | 0 | 0 | 0 | 1x10^30^ |
| W-jaws-non skin | 9x10^8^ | 5x10^4^ | 9x10^8^ | 0 | 0 | 0 | 0 | 1x10^30^ |
| W-MLC-non skin | 9x10^8^ | 5x10^4^ | 9x10^8^ | 0 | 0 | 0 | 0 | 1x10^30^ |
| W-target | 2x10^5^ | 5x10^4^ | 2x10^5^ | 1x10^-3^ | 1x10^-3^ | 2x10^5^ | 2x10^5^ | 6.3x10^-4^ |
| Cu-target | 2x10^5^ | 5x10^4^ | 2x10^5^ | 1x10^-3^ | 1x10^-3^ | 2x10^5^ | 2x10^5^ | 2x10^-2^ |

Note: E_abs_ was absorbed energy, C_1_ and C_2_ were the cutoff energy for elastic collisions; WCC and WCR are the cutoff energy for inelastic and bremsstrahlung interactions, respectively; and dsMax is an upper limit for the step length.

Supplementary table 2-The GPRs for PDD of 6MV photon beam.

| Field size (cm) | Gamma criterion | | | |
| --- | --- | --- | --- | --- |
|  | 3%/3mm | 2%/2mm | 1%/2mm | 1%/1mm |
| 3 | 100% | 99.33% | 99% | 99% |
| 4 | 100% | 100% | 99.33% | 99% |
| 6 | 100% | 99.33% | 99% | 99% |
| 8 | 100% | 100% | 99.67% | 99.33% |
| 10 | 100% | 100% | 99.33% | 99% |
| 15 | 100% | 100% | 100% | 99.33% |
| 20 | 100% | 100% | 100% | 99.33% |
| 30 | 100% | 100% | 100% | 99% |
| 40 | 100% | 100% | 99.33% | 99.33% |

Supplementary table 3-The GPRs for PDD of the 6MV FFF photon beam.

| Field size (cm) | Gamma criterion | | | |
| --- | --- | --- | --- | --- |
|  | 3%/3mm | 2%/2mm | 1%/2mm | 1%/1mm |
| 3 | 100% | 99.33% | 98.99% | 97.64% |
| 4 | 100% | 100% | 99.33% | 97.64% |
| 6 | 100% | 99.33% | 98.99% | 97.64% |
| 8 | 100% | 100% | 99.33% | 98.32% |
| 10 | 100% | 100% | 99.66% | 97.98% |
| 15 | 100% | 100% | 99.33% | 98.32% |
| 20 | 100% | 100% | 99.33% | 98.65% |
| 30 | 100% | 100% | 99.33% | 97.64% |
| 40 | 100% | 100% | 99.66% | 98.99% |

Supplementary table 4-The GPRs for OAR of the 6 MV photon beam.

| Field size(cm) | Gamma criterion | Depth (cm) | | | | |
| --- | --- | --- | --- | --- | --- | --- |
|  |  | 1.5 | 5 | 10 | 20 | 30 |
| 3 | 3%/3mm  2%/2mm | 100%  97.32% | 100%  98.66% | 100%  98.06% | 100%  98.2% | 100%  98.35% |
|  | 1%/2mm | 90.6% | 95.3% | 93.2% | 93.75% | 95.04% |
|  | 1%/1mm | 86.58% | 92.62% | 86.41% | 88.39% | 85.12% |
| 4 | 3%/3mm  2%/2mm | 100%  97.21% | 100%  97.2% | 100%  96.4% | 100%  97.56% | 100%  97.97% |
|  | 1%/2mm | 94.41% | 93.46% | 91.89% | 90.99% | 93.98% |
|  | 1%/1mm | 91.06% | 89.72% | 85.59% | 91.87% | 84.96% |
| 6 | 3%/3mm  2%/2mm | 100%  97.56% | 100%  96.85% | 100%  97.74% | 100%  98.62% | 100%  100% |
|  | 1%/2mm | 91.06% | 94.49% | 93.23% | 94.48% | 96.82% |
|  | 1%/1mm | 86.99% | 88.98% | 92.48% | 88.28% | 91.08% |
| 8 | 3%/3mm  2%/2mm | 100%  97.93% | 100%  97.32% | 100%  98.71% | 100%  100% | 100%  100% |
|  | 1%/2mm | 95.86% | 96.64% | 94.84% | 96.45% | 95.63% |
|  | 1%/1mm | 86.21% | 89.26% | 92.9% | 91.12% | 87.43% |
| 10 | 3%/3mm  2%/2mm | 100%  98.79% | 100%  98.82% | 100%  100% | 100%  100% | 100%  100% |
|  | 1%/2mm | 97.58% | 97.04% | 96.05% | 97.93% | 96.65% |
|  | 1%/1mm | 90.91% | 86.98% | 93.22% | 93.26% | 86.12% |
| 15 | 3%/3mm  2%/2mm | 100%  100% | 100%  100% | 100%  100% | 100%  100% | 100%  100% |
|  | 1%/2mm | 95.34% | 96.41% | 99.14% | 96.84% | 97.09% |
|  | 1%/1mm | 90.23% | 91.93% | 94.85% | 95.26% | 90.91% |
| 20 | 3%/3mm  2%/2mm | 100%  100% | 100%  100% | 100%  100% | 100%  100% | 100%  100% |
|  | 1%/2mm | 97.74% | 97.82% | 99.65% | 99.68% | 98.53% |
|  | 1%/1mm | 90.94% | 97.09% | 92.68% | 96.49% | 91.45% |
| 30 | 3%/3mm  2%/2mm | 100%  100% | 100%  100% | 100%  100% | 100%  100% | 100%  100% |
|  | 1%/2mm | 96.46% | 100% | 99.5% | 99.08% | 95.74% |
|  | 1%/1mm | 91.83% | 95.79% | 97.73% | 95.15% | 91.47% |
| 40 | 3%/3mm  2%/2mm | 100%  100% | 100%  100% | 100%  100% | 100%  100% | 100%  100% |
|  | 1%/2mm | 99.79% | 100% | 99% | 99% | 97.66% |
|  | 1%/1mm | 98.29% | 98.19% | 93.17% | 93.37% | 95.52% |

Supplementary table 5-The GPRs for OAR of 6MV FFF photon beam.

| Field size(cm) | Gamma criterion | Depth (cm) | | | | |
| --- | --- | --- | --- | --- | --- | --- |
|  |  | 1.5 | 5 | 10 | 20 | 30 |
| 3 | 3%/3mm  2%/2mm | 100%  97.32% | 100%  97.99% | 100%  97.03% | 100%  98.17% | 100%  97.48% |
|  | 1%/2mm | 93.96% | 93.96% | 91.09% | 93.58% | 94.96% |
|  | 1%/1mm | 93.29% | 93.96% | 88.12% | 88.09% | 86.55% |
| 4 | 3%/3mm  2%/2mm | 100%  95.15% | 100%  97.2% | 100%  96.4% | 100%  99.17% | 100%  98.47% |
|  | 1%/2mm | 89.32% | 95.33% | 90.99% | 92.56% | 95.42% |
|  | 1%/1mm | 87.38% | 91.59% | 88.29% | 90.09% | 87.02% |
| 6 | 3%/3mm  2%/2mm | 100%  96.75 % | 100%  97.64% | 100%  97.94% | 100%  98.64% | 100%  100% |
|  | 1%/2mm | 91.87% | 95.28% | 93.23% | 95.24% | 98.73% |
|  | 1%/1mm | 89.43% | 93.7% | 91.73% | 91.84% | 91.08% |
| 8 | 3%/3mm  2%/2mm | 100%  98.59% | 100%  100% | 100%  98.07% | 100%  100% | 100%  100% |
|  | 1%/2mm | 96.49% | 95.97% | 96.77% | 97.63% | 97.27% |
|  | 1%/1mm | 94.37% | 95.3% | 96.13% | 94.67% | 92.9% |
| 10 | 3%/3mm  2%/2mm | 100%  99.33% | 100%  100% | 100%  100% | 100%  100% | 100%  100% |
|  | 1%/2mm | 97.55% | 97.63% | 98.31% | 98.45% | 99.04% |
|  | 1%/1mm | 96.93% | 95.86% | 97.18% | 97.41% | 96.17% |
| 15 | 3%/3mm  2%/2mm | 100%  100% | 100%  100% | 100%  100% | 100%  100% | 100%  100% |
|  | 1%/2mm | 99.07% | 98.22% | 99.14% | 99.14% | 99.27% |
|  | 1%/1mm | 97.67% | 97.78% | 97.42% | 98.81% | 94.55% |
| 20 | 3%/3mm  2%/2mm | 100%  100% | 100%  100% | 100%  100% | 100%  100% | 100%  100% |
|  | 1%/2mm | 100% | 99.27% | 100% | 100% | 100% |
|  | 1%/1mm | 96.59% | 97.82% | 98.61% | 98.4% | 97.35% |
| 30 | 3%/3mm  2%/2mm | 100%  100% | 100%  100% | 100%  100% | 100%  100% | 100%  100% |
|  | 1%/2mm | 100% | 100% | 100% | 99.08% | 97.88% |
|  | 1%/1mm | 99.46% | 99.74% | 96.73% | 95.86% | 94.69% |
| 40 | 3%/3mm  2%/2mm | 100%  100% | 100%  100% | 100%  100% | 100%  100% | 100%  100% |
|  | 1%/2mm | 100% | 100% | 100% | 100% | 100% |
|  | 1%/1mm | 98.5% | 99.17% | 96.24% | 95.1% | 92.29% |

**DLG Optimization**

To minimize the difference of dose distributions, we also optimized DLG using 100 clinical treatment plans of different disease sites, including head and neck cancer (30%), thoracic cancer (30%), abdominal cancer (30%). There were also 10 stereotactic body radiation therapy (SBRT) treatment plans. To generate the treatment plans, photon optimizer (PO) algorithm was used. The resolution of PO optimization was set as 1.25mm for SBRT plans and 2.5mm for non-SBRT plans. The size of dose grid was set as 1mm for SBRT plans and 2mm for non-SBRT plans during final dose calculation using the Anisotropic Analytical Algorithm (AAA) algorithm that implemented in the Eclipse^TM^ version 13.6.23 (Varian Medical Systems Inc., Palo Alto, CA) was used to calculate the dose distributions. Gamma passing rate (GPR) was defined as the percentage of measurement points satisfying the condition of gamma index <1 using the gamma-index criteria (3% and 3mm, 2% and 2mm). In this study, the gamma-index criteria was 3 mm and 3% for non-SBRT treatment plans and 2mm and 2% for SBRT treatment plans. All the treatment plans were delivered in the mode of quality assurance. The dose distributions were measured using Delta4 phantom (Scandidos, Uppsala, Sweden). The optimized DLG was considered to generate a high GPR for all the treatment plans.
